# Hypoxia Promotes Mitochondrial Complex I Abundance via HIF-1α in Complex III and Complex IV Deficient Cells

**DOI:** 10.3390/cells9102197

**Published:** 2020-09-29

**Authors:** Amy Saldana-Caboverde, Nadee Nissanka, Sofia Garcia, Anne Lombès, Francisca Diaz

**Affiliations:** 1 Department of Neurology, University of Miami Miller School of Medicine, Miami, FL 33136, USA; asaldana@fiu.edu (A.S.-C.); n.nissanka@med.miami.edu (N.N.); sofiapgarcia@gmail.com (S.G.); 2 Institut Cochin, Unité U1016, INSERM, UMR 8104, CNRS, Université Paris 5, F-75014 Paris, France; anne.lombes@inserm.fr

**Keywords:** mitochondrial respiratory supercomplexes, oxidative phosphorylation, hypoxia, complex I, complex III, complex IV, Rieske iron sulfur protein, COX10, HIF-1α

## Abstract

Murine fibroblasts deficient in mitochondria respiratory complexes III (CIII) and IV (CIV) produced by either the ablation of *Uqcrfs1* (encoding for Rieske iron sulfur protein, RISP) or *Cox10* (encoding for protoheme IX farnesyltransferase, COX10) genes, respectively, showed a pleiotropic effect in complex I (CI). Exposure to 1–5% oxygen increased the levels of CI in both RISP and COX10 KO fibroblasts. De novo assembly of the respiratory complexes occurred at a faster rate and to higher levels in 1% oxygen compared to normoxia in both RISP and COX10 KO fibroblasts. Hypoxia did not affect the levels of assembly of CIII in the COX10 KO fibroblasts nor abrogated the genetic defect impairing CIV assembly. Mitochondrial signaling involving reactive oxygen species (ROS) has been implicated as necessary for HIF-1α stabilization in hypoxia. We did not observe increased ROS production in hypoxia. Exposure to low oxygen levels stabilized HIF-1α and increased CI levels in RISP and COX10 KO fibroblasts. Knockdown of HIF-1α during hypoxic conditions abrogated the beneficial effect of hypoxia on the stability/assembly of CI. These findings demonstrate that oxygen and HIF-1α regulate the assembly of respiratory complexes.

## 1. Introduction

The mitochondrial electron transport chain (ETC) and the oxidative phosphorylation (OXPHOS) system are fundamental to produce the majority of the cellular ATP. The ETC is located in the inner mitochondrial membrane (IMM) and is formed by four multi-subunit complexes (complexes I to IV, or CI to CIV) and two electron carriers, coenzyme Q (CoQ) and cytochrome *c*. The ETC multimeric complexes are formed by subunits encoded by both the nuclear and the mitochondrial genome, with the exception of CII, which is entirely encoded by the nucleus. The proton pumping complexes of the ETC (CI, CIII and CIV) can form associations with each other. These supramolecular arrangements are termed supercomplexes (SCs). The first evidence of respiratory complex’s interactions was provided by Shägger and Pfeiffer [1] and Cruciat [2] almost two decades ago using mild extraction conditions and blue-native polyacrylamide gel electrophoresis.

Mitochondrial SCs have been described in multiple organisms including bacteria, plants, fungi and mammals. The composition of SCs varies depending on the organism, the tissue and the metabolic state. Mammalian SCs are mainly formed by associations of CIII_2_ + CIV, CI + CIII_2_, CI + CIII_2_ + CIV and CI + CIII_2_ + CIV_2−n_. The CI + CIII_2_ + CIV arrangement is named the “respirasome”. There are also higher order arrangements of SCs forming respiratory strings [3] and other high molecular weight architectures (CI_2_ + CIII_2_ + CIV_2−n_), termed megacomplexes [4]. SCs are capable of respiration and the transfer of electrons from NADH to oxygen [5]. The precise organization of CI, CIII and CIV in SCs has recently been elucidated using single-particle cryo-electron microscopy [4,6,7,8,9].

During the last decade, our knowledge of SCs has increased exponentially. However, our understanding of how the SCs are assembled, how they are regulated and what their physiological function in health and disease is remains limited (reviewed in [10,11,12,13]). Various factors described to aid in SCs formation include: Rcf1 and Rcf2 [14,15,16]; COX7A2L [17,18,19,20]; Cardiolipin [21]; HIGD2A [16,22,23]; MCJ/DnaJC15 [24]; and prohibitins [25]. In addition, mitochondrial dynamics and cristae morphology also have been reported to play a role in SC assembly [26,27,28].

One of the functions attributed to SCs is to facilitate a more efficient electron flow by supporting substrate channeling and reducing the formation of free radicals [17,20,29,30,31]. In the ETC, CI and CIII are the most prominent sites for the production of reactive oxygen species (ROS) [32]. Previous studies showed that disruption of SC CI + CIII_2_ increased ROS production, suggesting that one of the functions of SCs is to prevent the formation of excessive free radicals during electron flux [33]. On the other hand, increases in oxidative stress can cause the instability of respiratory complexes, particularly of CI, and subsequently impact the stability of SCs. Supporting this notion are our studies in mouse fibroblasts and in the neuron-specific Rieske iron sulfur protein (RISP) KO mouse [34,35,36]. We showed that in the neuronal specific RISP KO mouse, high levels of oxidative stress in the brain, particularly in the piriform cortex, compromised the stability of respiratory complexes and SC formation. Treatment of mice with the mitochondrial-targeted antioxidant MitoTEMPO increased the stability of CI and SCs in piriform cortex [36]. In mouse neurons, CI is mainly found in SCs, whereas in astrocytes, CI is predominantly free [31]. The free CI in astrocytes was associated with a higher production of free radicals. Overexpression of NDUFS3 (CI subunit abundantly expressed in neurons) in astrocytes promoted the association of CI into SCs and reduced the levels of ROS [31].

Another physiological role attributed to SCs is the stabilization/assembly of its individual components [37,38,39]. We and others reported that CIII and CIV were necessary for maintenance of CI in mammalian mitochondria [35,40,41,42,43,44]. In further studies, using fibroblasts lacking either RISP (catalytic subunit of CIII) or protoheme IX farnesyltransferase, COX10 (assembly factor of CIV), we found that the pleiotropic effect on CI was mitigated when cells were exposed to low oxygen levels, suggesting a ROS-dependent mechanism affecting CI assembly/stability in normoxic conditions [34,35]. Interestingly, chronic exposure to mild hypoxia was beneficial for the treatment of a mouse model of CI deficiency (Ndufs4^−/−^) [45,46]. Even though the genetic defect still existed, 11% hypoxia significantly extended the life span and ameliorated disease phenotype in the Ndufs4^−/−^ mouse [45,46]. Activation of the hypoxic transcriptional program involves the stabilization of hypoxia inducible factor 1 subunit alpha (HIF-1α), which, when binding to the HIF-1β subunit, forms the active transcription factor HIF-1. This transcription factor binds to hypoxic response elements (HRE) in the genome and regulates the expression of metabolic genes [47].

In this study we investigate in more detail the beneficial effect of hypoxia on the pleiotropic effect of CI observed in CIII and CIV deficiencies in various human and mouse cell lines with mutations in either the nuclear genome or the mitochondrial DNA (mtDNA). Given the important role of the hypoxic transcriptional program in regulating mitochondrial function, we examined the involvement of HIF-1α in the stability/assembly of CI during hypoxia. We found that HIF-1α was required for the assembly of CI in RISP and COX10 KO fibroblasts. Our results show that oxygen levels regulate the assembly of respiratory complexes and provide some mechanistic insight into how mild hypoxia ameliorates the phenotype in the CI deficient mouse.

## 2. Materials and Methods

### 2.1. Cell Culture

In this study, we used various murine and human cell lines. The RISP KO lung fibroblast (clone #8.5), the COX10 KO skin fibroblast (clone #19) and their respective controls were created and characterized by us in [35,42]. Human fibroblasts derived from Patient “D” and Patient “N” with defects in CIV and CIII, respectively, were described by us [48,49]. The cybrid cell line with a mutation in Cox1 (CA65 cybrid) was described in [50] and the Cytb 17.3E cybrid carrying a 4 bp deletion in the cytochrome b gene was described in [51]. All cell lines were grown in high glucose Dulbecco’s modified Eagle’s medium (DMEM, GIBCO Cat#11965, ThermoFisher Scientific, Waltham, MA, USA) supplemented with 10% fetal bovine serum (Seradigm Cat# 97068-085, VWR, Radnor, PA, USA), 1 mM Pyruvate (GIBCO Cat#11360070, Thermo Fisher Scientific, Waltham, MA, USA, 50 μg/mL uridine (Sigma U3003, St Louis, MO, USA) and 20 μg/mL gentamicin (GIBCO Cat# 15750060, ThermoFisher Scientific, Waltham, MA, USA) in a 5% CO_2_ incubator at 37 °C.

Patient cells were obtained with signed informed consent and are not traceable to the original patient. All subjects gave their informed consent before giving a skin biopsy for fibroblasts derivation to be used in diagnostic procedures and medical research without time limitation. The study was conducted in accordance with the Declaration of Helsinki. Informed consent was approved by the Institutional Review Board Ethical Committee of La Salpêtrière Hospital, Paris in 1997. Animal work was approved by the University of Miami Institutional Animal Care and Use Committee (AICUC, protocol number 10-205). We obtained the approval by the Institutional Animal Care and Use Committee (IACUC) at the University of Miami. The University of Miami has an Animal Welfare Assurance on file with the Office of Laboratory Animal Welfare (OLAW), National Institutes of Health. The assurance number is #A-3224-01, effective 11 July 2007 and approved through 30 November 2023. Additionally, as of 11 July 2007, The University of Miami AICUC committee has full accreditation with the Association for Assessment and Accreditation of Laboratory Animal Care (AAALAC International), site 001069, latest effective date 8 October 2019. Our approval for use of animals for research was under protocol number 10-205, which was originally approved in 2 September 2010. The protocol has been undergoing revision and approval by IACUC annually and is renewed every 3 years.

### 2.2. Normoxic and Hypoxic Conditions

The normoxic conditions consisted of regular tissue culture conditions of an atmosphere of 21% O_2_ and 5% CO_2_. To create the hypoxic conditions, cells were placed in a chamber at 37 °C connected to a Proox-110 oxygen controller (BioSpherix Reming Bioinstruments, Parish, NY, USA) hook to a nitrogen-CO_2_ mixture tank (Mediblend clinical blood gas mixture 5% CO_2_ plus nitrogen, Airgas South, Fairburn, GA, USA) to produce a hypoxic atmosphere of desired oxygen concentrations from 1% to 5% O_2_. Cells (2–3 × 10^6^) were plated in 10 cm dishes and immediately placed in the CO_2_ incubator (normoxia) or in the hypoxic chamber and exposed to these conditions for 24 h or longer times (up to 96 h) depending on the experiment. The cells were immediately collected by trypsinization after different oxygen exposures, washed in PBS and cell pellets stored at −80 °C until analyzed.

### 2.3. Doxycycline Treatment to Deplete Mitochondrial Respiratory Complexes

To deplete mitochondria of assembled respiratory complexes, cells were treated with 15 μg/mL doxycycline (Sigma D9891, St Louis MO, USA) for 6 days as described before [52,53]. Media containing doxycycline was changed daily. After 6 days, doxycycline was removed and cells left to recover either in hypoxia (1% O_2_) or normoxia conditions in regular growth media for different times 0, 24, 48, 72 and 96 h (T0, T24, T48, T72 and T96). Cells at the different time points were collected by trypsinization, washed in PBS and pellets stored at −80 °C until analyzed.

### 2.4. Determination of Mitochondrial ROS Production Using Redox Sensitive GFP

To measure the production of ROS in hypoxia, we used the ratiometric redox-sensitive biosensor RoGFP (redox sensitive GFP) targeted to either the mitochondrial matrix or the inter membrane space [54]. The advantage of using this probe is that the oxidized and reduced state of the protein is measured at two excitation wavelengths, 405 and 495 nm, and emission at 510 nm independently of the expression levels. The ratio of fluorescent 405/495 is directly proportional to the levels of ROS [54]. Plasmids (1 μg) expressing the targeted RoGFP to either the mitochondria matrix (matrix-RoGFP, Addgene plasmid #49437, Watertown, MA, USA) or the intermembrane space (GPD-RoGFP, Addgene plasmid #49436, Watertown, MA, USA) [54] were used to transfect controls, RISP and COX10 KO fibroblasts using the GenJET transfection reagent (SignaGen Laboratories, Frederick, MD, USA) as per manufacturer instructions. After 24 h of transfection, cells were exposed to either normoxia or 1% oxygen for 24 h. After hypoxia incubation, cells were fixed inside the hypoxic chamber with 4% PFA for 15 min and washed with PBS 3 times as previously described [55]. Fixing cells was performed to avoid changes in fluorescence due to re-oxygenation after hypoxic treatment when processing cells for microscopy. Fluorescence 405/495 ratio was the same in cells fixed or not fixed under normoxic conditions, and fluorescence did not change in fixed cells after the addition of 10 mM DTT (Sigma D9779, St Louis, MO, USA) or 10 mM tBOH (Sigma B2633, St Louis, MO, USA). Fluorescence was recorded using a Leica SP5 multiphoton upright confocal microscope at two excitation wavelengths of 405 and 495 nm and emission wavelength of 510 nm. Fluorescent intensities at the two excitation wavelengths for each cell were calculated using ImageJ software and the ratio 405/495 was calculated as a measurement of redox state of the cells. Fluorescence ratios for at least 20–30 cells per experiment were imaged and independent experiments were done at least 3 times. Data from all independent experiments were combined.

### 2.5. Blue Native Gel Electrophoresis, SDS-Polyacrylamide Gel Electrophoresis and Western Blotting

To analyze the assembly of OXPHOS supercomplexes, we used blue native gel electrophoresis (BN-PAGE). Cell pellets obtained after normoxia or hypoxia incubation were resuspended in 1.5 M amino caproic buffer (80–100 μL). Supercomplexes were solubilized with digitonin at a protein to detergent ratio of 1:8 (*wt*/*wt*), as described in [36]. About 15–20 μg of proteins were separated in 3–12% acrylamide Bis-Tris precast native gels (Invitrogen Cat# BN1001BOX and BN1003BOX, ThermoFisher Scientific, Waltham, MA, USA) and proteins transferred to PVDF membranes for Western blotting as described in [56]. PVDF membranes were blocked with 5% milk and sequentially blotted with antibodies against different subunits of respiratory complexes. The signal was developed using ECL and acquired either by exposure to X-ray film or using the ChemiDoc imaging system (Bio-Rad, Hercules, CA, USA). VDAC1 and Tim23 were used as mitochondrial loading controls.

For SDS-PAGE, 20 μg of protein were heated at 70 °C for 15–20 min and separated in 4–20% acrylamide “Stain Free” precast gels (Bio-Rad Cat# 4561094). After electrophoresis proteins were transferred to PVDF membranes and total proteins were visualized by the stain free technology using the ChemiDoc system. Then membranes were processed as described above for BN-PAGE.

We used the following primary antibodies: NDUFA9 (Cat# ab14713), UQCRC1 (Cat# ab110252), UQCRC2 (Cat# ab14745), ATP5A (Cat# ab14748), COX1 (Cat# ab14705), SDHA (Cat# ab14715) and VDAC1 (Cat# ab14734) from Abcam (Cambridge, MA, USA); Tim23 (Cat# 611222) from BD Bioscience (San Jose, CA, USA); HIF-1α antibodies were obtained from Novus (Cat# NB100-105, Centennial, CO, USA) or from Cell Signaling Technology (Cat# 36169, Danvers, MA, USA); tubulin (Cat# T9026) and actin (Cat# A2066) from Sigma (St Louis, MO, USA). All antibodies were used at a 1/1000 dilution with the exception of tubulin and actin that were used at 1/20,000 dilution. Secondary anti-mouse (Cat# 7076) and anti-rabbit (Cat# 7074) IgG Horseradish Peroxidase labeled antibodies were obtained from Cell Signaling Technology (Danvers, MA, USA) and used at a 1/2000 dilution.

Optical density of immunoreactive bands in Western blots were measured using the ImageJ software.

### 2.6. HIF-1α SiRNA Silencing

COX10 and RISP fibroblasts were transfected with either a non-targeted control or with a mixture of 3 Stealth RNAi duplex oligoribonucleotides against mouse HIF-1α. The oligonucleotides were obtained from ThermoFisher Scientific (Cat# MSS2055124, MSS205125 and MSS205126, Waltham, MA, USA). The sequences of the RNA oligonucleotide pairs were for Cat# MSS2055124: 5′-GGG CCGCUCAAUUUAUGAAUAUUAU-3′ and 5′-AUAAUAUUCAUAAAUUGAGCGGCCC-3′; for Cat# MSS205125: 5′-UACUCAGAGCUUUGGAUCAAGUUAA-3′ and 5′-UUAACUUGAUCCAAAGCUCUGAGUA-3′; and for Cat# MSS205126: 5′-GAAAGAAUUACUGAGUUGAUGGGUU-3′ and 5′-AACCCAUCAACU CAGUAAUUCUUUC-3′. Cells were plated at ~1–2 × 10^5^ cells/well in 12-well plates and next day transfected with siRNA oligos at 10 nM using 3 μL DharmaFECT transfection reagent (Dharmacon, Lafayette, CO, USA) as described in the manufacturer’s specifications. Twenty-four hours post transfection, cells were confluent and were subsequently transferred to 6 well plates. Seventy-two hours after transfection, cells were split into two 6-well plates for each cell line. One of the plates underwent a second round of transfection with HIF-1α siRNA oligoribonucleotides or with a non-targeted control. The plate with only one round of transfection was placed under hypoxia (1% oxygen) for 24 h) and cells were collected for BN- and SDS-PAGE analysis (120 h after first transfection). The plate with 2 rounds of transfection was split into two 6-well plates and one of them was exposed to 1% oxygen for 24 h and cells collected for analysis at 144 h after first transfection and 72 h after second transfection.

To assess the efficiency of silencing HIF-1α with the siRNA oligos, we performed Western blots. Briefly, siRNA transfected cells were exposed to hypoxia (1% oxygen) for 24 h and immediately lysed with 100 μL of 2× Laemmli sample buffer without bromophenol blue. Proteins were quantified with DC protein kit from Bio-Rad (Cat# 5000112, Hercules, CA, USA) and then separated in SDS-PAGE using precast 10% acrylamide gels (Bio-Rad Cat# 4561034, Hercules, CA, USA). Proteins were transferred to PVDF membranes (Bio-Rad Cat#1620264, Hercules, CA, USA) using the Trans-Blot Turbo transfer system (Bio-Rad, Hercules, CA, USA) and blotted with HIF-1α and tubulin antibodies.

### 2.7. Statistical Analysis

Graphs and statistical analysis were performed using GraphPad Prism 8 software (San Diego, CA, USA). Statistical differences were determined using Student *t*-test or one-way analysis of variance (ANOVA) followed by Tukey’s multiple comparisons test. Differences were considered statistically significant when *p* values were * *p* < 0.05; ** *p* < 0.01; *** *p* < 0.001 and **** *p* < 0.0001. The results are presented as mean ± standard deviation (SD), and sample size (*n*) is indicated in the figure legend. Most of the data were obtained from 2 to 3 independent experiments, with the exception of Patient D (Pat.D) and Patient N (Pat.N) fibroblasts and CA cybrids, and indicated in their respective figure legends as a sample size of *n* = 1. Other results are also indicated as *n* = 1 (doxycycline experiments), even though they were performed at least 2 times, because the optical densities of the blots were too different (after corresponding normalization for protein loading) to allow any comparison.

## 3. Results

### 3.1. Stabilization of CI by Different Oxygen Concentrations in CIII and CIV Deficient Cells

**Figure 1:**
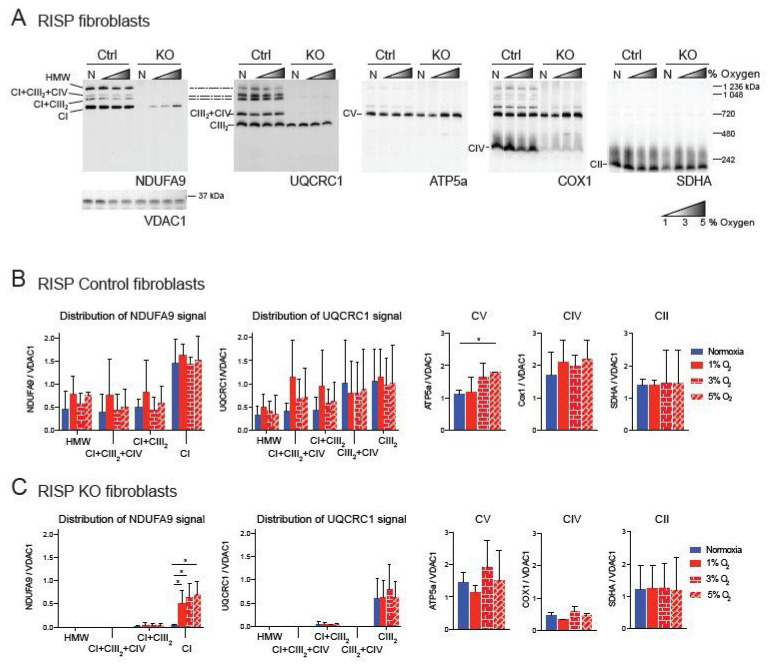
Blue native gel electrophoresis of Rieske iron sulfur protein (RISP) murine fibroblasts exposed to different oxygen concentrations. (**A**) RISP control and KO lung fibroblasts were exposed to normoxia (N, 21% oxygen), 1, 3 and 5% oxygen for 24 h. Mitochondrial proteins were extracted with digitonin and oxidative phosphorylation (OXPHOS) complexes and supercomplexes (SCs) analyzed by blue native gel electrophoresis (BN-PAGE) followed by Western blot using antibodies against respiratory complex subunits. Shaded triangles represent hypoxic conditions of increasing oxygen concentrations (1, 3 and 5% oxygen). Antibodies were sequentially added in two identical blots in the following order, in blot 1: NDUFA9 (complex I (CI) subunit), ATP5A (complex V (CV) subunit) and COX1 (complex IV (CIV) subunit) and SDHA (complex II (CII) subunit), in blot 2: UQCRC1 (complex III (CIII) subunit). The positions of each respiratory complex (CI-CV) and SCs, as well as molecular weights, are indicated in the figure. SCs include high molecular weight (HMW), CI + CIII_2_ + CIV, CI + CIII_2_, and CIII_2_ + CIV architectures. (**B**) Quantification of antibody signals and distribution into complexes and SCs of control lung fibroblasts shown in (**A**) of NDUFA9, UQCRC1, ATP5A, COX1 and SDHA normalized to the mitochondrial loading control VDAC1. Optical density was calculated using the ImageJ software. Graphs represent mean and standard deviation (*n* = 3). Statistical differences among groups was calculated using one-way ANOVA analysis followed by Tukey’s multiple comparisons test where * *p* < 0.05 was considered statistically significant. Blue bars represent normoxia, red bars represent 1% O_2_, red horizontal brick pattern bars represent 3% O_2,_ and red diagonal brick pattern bars represent 5% O_2_. (**C**) Quantification of antibody signals in RISP KO fibroblasts (*n* = 3) as described in (**B**). Antibodies were sequentially added on two identical blots in the following order, on blot 1: NDUFA9, ATP5A, COX1, and SDHA, on blot 2: UQCRC1 and SDHA. Exposure to 1–3% oxygen increased the levels of CI and CI + CIII_2_ in COX10 KO fibroblasts. Exposure to hypoxia significantly increased the levels of CI in RISP KO fibroblasts.

**Figure 2:**
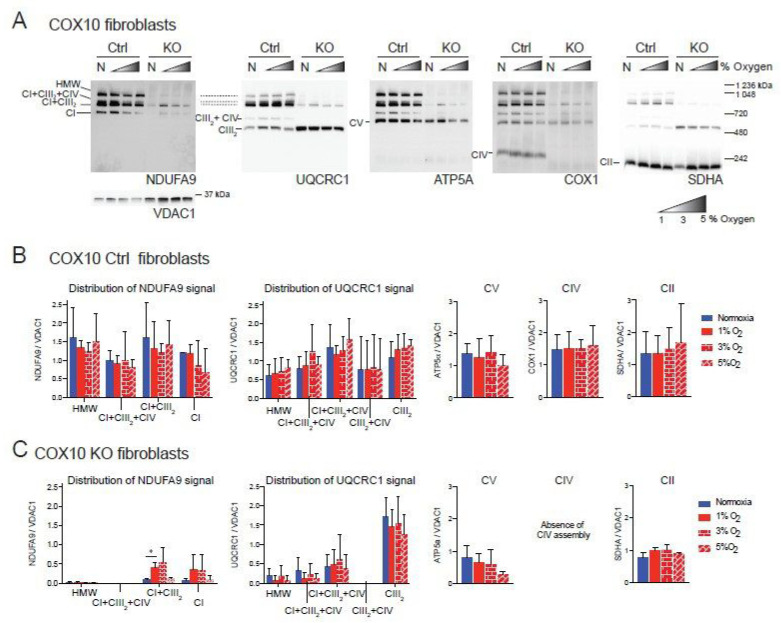
Blue native gel electrophoresis of COX10 murine fibroblasts exposed to different oxygen concentrations. (**A**) COX10 control and KO skin fibroblasts were exposed to normoxia (N, 21% oxygen), 1, 3 and 5% oxygen for 24 h. Mitochondrial proteins were extracted with digitonin and complexes and SCs analyzed by BN-PAGE followed by Western blot using antibodies against respiratory complex subunits. Shaded triangle represents hypoxic conditions of increasing oxygen concentrations (1, 3 and 5% oxygen). The positions of each respiratory complex (CI-CV) and SCs, as well as molecular weights, are indicated in the figure. SCs include high molecular weight (HMW), CI + CIII_2_ + CIV, CI + CIII_2_ and CIII_2_ + CIV architectures. (**B**) Quantification of antibody signals of control lung fibroblasts shown in (**A**) of NDUFA9 (CI subunit), UQCRC1 (CIII subunit), ATP5A (CV subunit), COX1 (CIV subunit) and SDHA (CII subunit) normalized to the mitochondrial loading control VDAC1. Optical density of antibody signals in blots was calculated using the ImageJ software. (**C**) Quantification of antibody signals in RISP KO fibroblasts as indicated in (**B**). Graphs represent mean and standard deviation (*n* = 2). Statistical significance among groups was determined by one-way ANOVA analysis followed by a Tukey’s multiple comparisons test where * *p* < 0.05 was considered statistically significant. Blue bars represent normoxia, red bars represent 1% O_2_, red horizontal brick pattern bars represent 3% O_2,_ and red diagonal brick pattern bars represent 5% O_2_. Antibodies were sequentially added on two identical blots in the following order, on blot 1: NDUFA9, ATP5A and COX1, on blot 2: UQCRC1 and SDHA. Exposure to 1–3% oxygen increased the levels of CI and CI + CIII_2_ in COX10 KO fibroblasts.

We have previously shown a pleiotropic effect on the stability of CI in mouse fibroblasts deficient in RISP and in COX10 [35,42]. This pleiotropic effect was abrogated by exposure to hypoxia (1% oxygen) [34,35]. Because under physiological conditions oxygen tensions in tissues are around 3%, we decided to investigate the effect of different concentrations of oxygen on CI stability. Controls, RISP and COX10 KO fibroblasts were exposed to either normoxia (21% oxygen) or to 1%, 3% and 5% oxygen for 24 h and respiratory complexes and supercomplexes (SCs) were analyzed by blue native polyacrylamide gel electrophoresis (BN-PAGE) followed by Western blot (Figure 1 and Figure 2). In RISP control lung fibroblasts, the CI subunit NDUFA9 signal was found to be distributed in different SC arrangements including high molecular weight (HMW), CI + CIII_2_ + CV, CI + CIII_2_ and free CI at a proportion of 31, 5, 13 and 51%, respectively (Figure 1A, NDUFA9 panel). The CIII subunit UQCRC1 was distributed into SCs of HMW, CI + CIII_2_ + CIV, CI + CIII_2_, CIII_2_ + CIV and free CIII at a proportion of 6, 10, 10, 30 and 44%, respectively (Figure 1A, UQCRC1 panel). Hypoxia did not alter the levels of UQCRC1 nor its distribution into the different CIII architectures (Figure 1A,B center left graph). No other differences were observed in OXPHOS complexes in the RISP control lung fibroblasts with the exception of CV, which was significantly increased at 5% oxygen compared to normoxia (Figure 1A, ATP5A panel and 1B, graph center right). In RISP KO lung fibroblasts, 1–5% oxygen conditions favored the stability of CI and significantly increased the levels of NDUFA9 signal (normalized to the mitochondrial loading control VDAC1) about 15–20-fold compared to normoxia levels (Figure 1A,C). The NDUFA9 signal was found mainly as free CI (92–95%) and a very small proportion as SC CI + CIII_2_ (5–8%). The UQCRC1 signal was mainly found as free CIII and very little was detected in SC CI + CIII_2_ (~5%). Hypoxia exposure did not alter the levels or distribution of UQCRC1 and no significant differences were observed between the levels of the other OXPHOS complexes between normoxia (Figure 1A UQCRC1, ATP5A, COX1 and SDHA panels, respectively, and Figure 1C graphs) and the different oxygen conditions in RISP KO fibroblasts.

Similar result were observed in the COX10 skin fibroblasts. In COX10 control skin fibroblasts, the NDUFA9 signal was distributed into free CI and SCs as in the lung RISP fibroblast although the proportion was different. The control COX10 fibroblasts had higher levels of NDUFA9 signal in SCs of HMW, CI + CIII_2_ + CI and CI + CIII_2_ and less of free CI at a proportion of 32, 17, 33 and 18%, respectively (Figure 2A, NDUFA9 panel and Figure 2B). Similarly, the UQCRC1 signal was found in SCs of HMW, CI + CIII_2_ + CI and CI + CIII_2,_ CIII_2_ + CIV and free CIII at a proportion of 11, 16, 48, 4 and 21% respectively (Figure 2A, UQCRC1 panel and Figure 2B). Exposure to different levels of oxygen did not alter the level of any of the OXPHOS complexes (Figure 2A,B). In the COX10 KO fibroblasts, increased levels of CI and SC CI + CIII_2_ (NDUFA9 signal) were observed at 1–3% oxygen, although differences only reached statistical significance for SC CI + CIII_2_ at 1% oxygen (Figure 2A, NDUFA9 panel and Figure 2C, left graph). Unlike in the RISP KO lung fibroblasts, in the COX10 KO fibroblasts the NDUFA9 signal was found mainly in SC CI + CIII_2_ (~85%) rather than as free CI (~15%). The UQCRC1 signal was found mainly as free CIII and less in SCs (~80 and 20% normalized to VDAC1, respectively). Hypoxia did not alter the levels and distribution of the UQCRC1 signal, nor the levels of CV and CII (Figure 2A,C). The CIII_2_ + CIV arrangement was not observed in the COX10 KO cells (Figure 2A, UQCRC1 panel and Figure 2C center left graph) due to the lack of CIV assembly (Figure 2A, COX1 panel).

**Figure 3:**
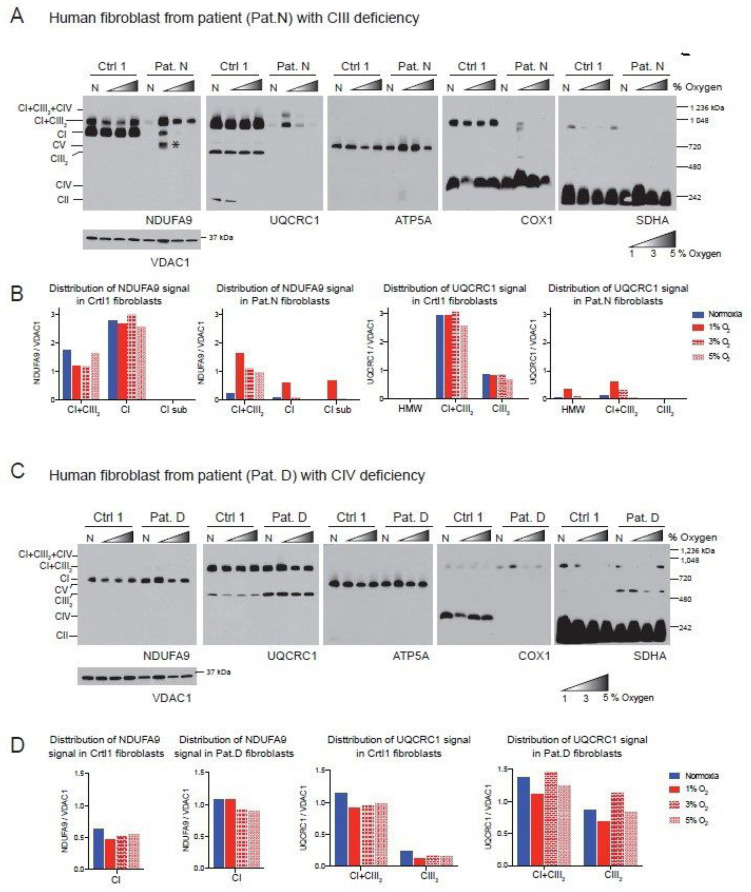
Effect of hypoxia in the stability of CI in patient fibroblasts with defects in CIII and CIV. (**A**) Control 1 and Patient N (Pat.N) fibroblasts with a defect in CIII caused by a mutation in the *CYC1* gene were exposed to normoxia (N, 21% oxygen), 1, 3 and 5% oxygen for 24 h. Mitochondrial proteins were extracted with digitonin and OXPHOS complexes and SCs analyzed by BN-PAGE followed by Western blot using antibodies against respiratory complex subunits. Shaded triangles represents increasing oxygen concentration (1, 3 and 5% oxygen). The positions of each respiratory complex and SC, as well as molecular weights, are indicated in the figure. Pat.N exhibited a pleiotropic effect in CI in normoxia and no assembly is observed. When exposed to 1% oxygen, CI assembly is stabilized and an assembly intermediary (* of about >720 kDa), fully assembled CI and SC CI + CIII_2_ can be observed in the NDUFA9 panel. At higher oxygen concentrations, CI was observed only in SC CI + CIII_2_. CIII assembly, which is defective in Pat.N, is also stabilized by exposure to low oxygen and CIII and found in CI + CIII_2_ arrays and not as a free complex in the UQCRC1 panel. (**B**) Densitometry and distribution of antibody signals of control and patient fibroblasts of blots shown in A (*n* = 1) for NDUFA9 (CI subunit) and UQCRC1 (CIII subunit) normalized to the mitochondrial loading control VDAC1. Optical density of antibody signals on the blots was calculated using the ImageJ software. (**C**) Control 1 and Patient D (Pat.D) fibroblasts with a defect in CIV caused by a mutation in SURF1 were exposed to different oxygen concentrations as indicated in (**A**), *n* = 1. (**D**) Densitometry and distribution of antibody signals of control and Pat.D fibroblasts of blots shown in C (*n* = 1) for NDUFA9 and UQCRC1 normalized to the mitochondrial loading control VDAC1 as described in (**B**). Blue bars represent normoxia, red bars 1% O_2_, red horizontal brick pattern 3% O_2_ and red diagonal brick pattern 5% O_2_. Antibodies were sequentially added on two identical blots in the following order, on blot 1: NDUFA9, COX1 ATP5A and on blot 2: UQCRC1 and SDHA.

To determine the universality of the positive effect of hypoxia on the ETC, we analyzed two human fibroblast cell lines with CIII and CIV deficiency that we had characterized previously [48]. The first of these cell lines was derived from Patient N (Pat.N) who had an isolated CIII deficiency due to a cytochrome *c_1_* (CYC1) mutation [48,49], impairing the assembly of CIII (Figure 3A, Lane N, UQCRC1 panel). In addition to the CIII defect, BN-PAGE revealed a pleiotropic defect in the assembly of CI (Figure 3A, lane N, NDUFA9 panel). When Pat.N fibroblasts were exposed to hypoxia, an increase in the levels of NDUFA9 normalized to VDAC1 was observed (Figure 3A,B). In Pat.N, the NDUFA9 signal was found in bands corresponding to SC CI + CIII_2_, free CI and an assembly intermediate above 720 kDa (Figure 3A, CI assembly intermediate depicted with asterisk in NDUFA9 panel and named CI-sub in Figure 3B, graph in center left). Interestingly, hypoxia exposure also increased the UQCRC1 signal (normalized to VDAC1), which was associated with SC CI + CIII_2_ and with the respirasome CI + CIII_2_ + CIV in Pat.N fibroblasts (Figure 3A, UQCRC1 panel and Figure 3B, graph on the right). The best stabilizing effect on CI and CIII in Pat.N fibroblasts was observed at 1% oxygen.

The second human fibroblast tested was obtained from Patient D (Pat.D) with mutations in the *SURF1* gene resulting in a defect in CIV assembly (Figure 3C, Cox1 panel) [48]. Interestingly, the COX1 signal was observed in SCs, suggesting that the *SURF1* mutant fibroblasts still can produce low levels of CIV, which most likely is more stable when associated with the respirasome. Unlike the mouse COX10 KO fibroblasts, Pat.D did not have a pleiotropic defect in the assembly of CI. Indeed, Pat.D fibroblasts had about 1.7-fold higher NDUFA9 signal than control fibroblasts (normalized to VDAC1 signal) and even higher levels of CIII detected with the UQCRC1 antibody (Figure 3D). Exposure to 1% oxygen did not increase the levels of CI in Pat.D (when normalized to VDAC1 loading) and 3 and 5% oxygen produced a rather slight decrease in CI (Figure 3C, NDUFA9 panel and Figure 3D center left graph).

Because 1% oxygen resulted in an improved CI assembly/stability in most cell lines tested, we decided to use this oxygen concentration in subsequent experiments.

### 3.2. Hypoxia Increased the Levels of CI in CYTB Cybrids and Caused Redistribution of CI in COX1 Cybrids

We also examined CI assembly in cybrid cell lines with primary CIII and CIV deficiency. In cybrids, the genetic defect is restricted to the mtDNA, in contrast to the above described cells, where the genetic defect resides in the nuclear DNA. The two transmitochondrial cybrids analyzed were the Cytb 17.3E and the CA65 [50,51].

**Figure 4:**
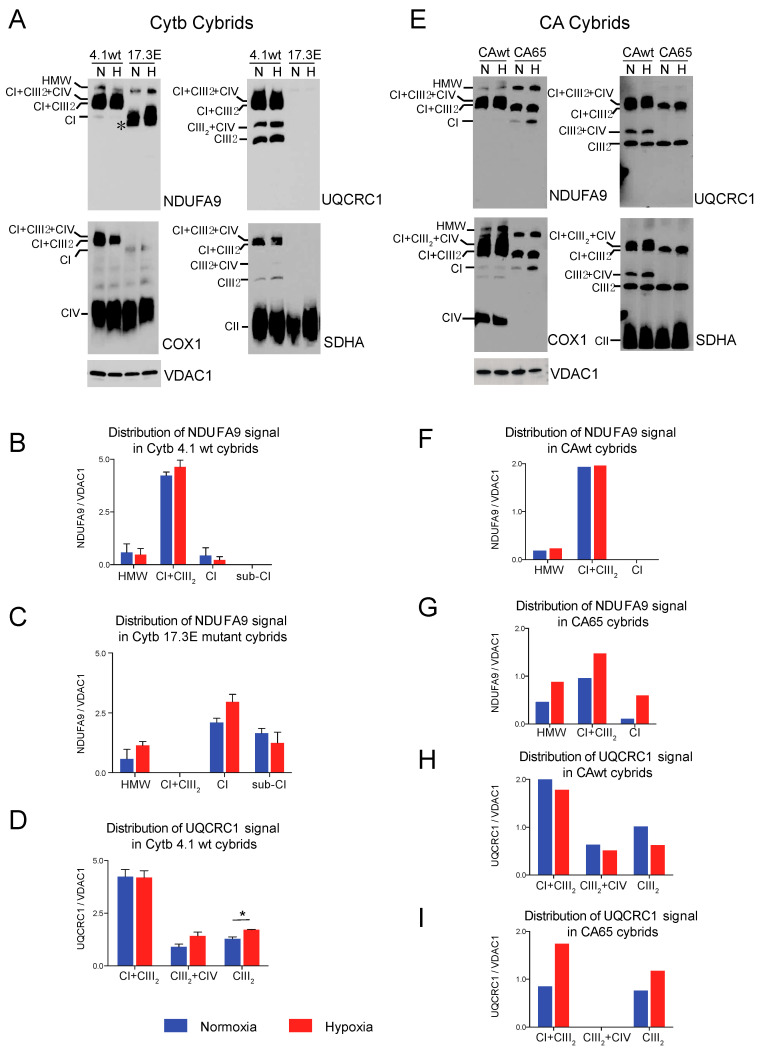
Effect of hypoxia in stability of respiratory complexes in human cybrids with defects in CIII and CIV. (**A**) Cytochrome b cybrids: control wild type (Cytb 4.1wt) and cybrid carrying a homoplasmic 4 base pair deletion in the *CYTB* gene of the mtDNA (Cytb 17.3E) were exposed to normoxia (N, 21% oxygen) or hypoxia (H, 1% oxygen) for 24 h and mitochondrial proteins were extracted with digitonin and OXPHOS complexes and SCs analyzed by BN-PAGE followed by Western blot using antibodies against respiratory complex subunits. The positions of each respiratory complex, SCs (HMW, CI + CIII_2_ + CIV, CI + CIII_2_, CIII_2_ + CIV) and molecular weight markers are indicated in the figure. Cytb 17.3E cybrids in normoxia display a fast migrating band with the NDUFA9 antibody corresponding to an assembly intermediate (* or Sub-CI) of about 830 kDa, when exposed to hypoxia this band migration is retarded indicating the presence of fully assembled CI. (**B**,**C**) Quantification and distribution of NDUFA9 (CI subunit) and in 4.1wt and 17.3E cybrids normalized to mitochondrial loading control VDAC1. (**D**) UQCRC1 (CIII subunit) signal of 4.1wt cybrids normalized to VDAC1. Optical densities of antibody signals in the blots were calculated using ImageJ software. Graphs represent mean and standard deviation (*n* = 2). Statistical differences between normoxia and hypoxia were determined using Student’s *t*-test where statistical significance is indicated by * *p* < 0.05. Blue bars represent normoxia and red bars 1% oxygen. (**E**) CA cybrids are CIV deficient and carry a mutation in the Cox1 gene. CAwt cells do not have any mutation in the mtDNA whereas CA65 carry the Cox1 mutation at a 65% heteroplasmy. Cells were exposed to hypoxia as described in (**A**). (**F**–**G**) Densitometry of blots shown in (E) for NDUFA9 panel normalized to VDAC1 (*n* = 1). (**H**–**I**) UQCRC1 signal in (E) normalized to VDAC1 (*n* = 1). Blue bars indicate normoxia and red bars 1% oxygen. Antibodies were sequentially added in two identical blots in the following order, in blot 1: NDUFA9 and COX1 (CIV subunit) and in blot 2: UQCRC1 and SDHA (CII subunit). In CA65 cybrids, there is no a pleiotropic effect on CI and hypoxia increased the level of free CI and SCs of HMW and CI + CIII_2_.

The Cytb 17.3E cybrid contains an out-of-frame 4 bp deletion in the 5′ end of the apocytochrome b gene (*CYTB*) [51]. The Cytb 17.3E cybrid is homoplasmic (100% mutant mtDNA) for the micro deletion that resulted in the absence of Cytb and subsequent defect in CIII assembly (Figure 4A, UQCRC1 panel). As a control, we used the Cytb 4.1wt cybrid containing 100% wild type mtDNA. Both control and mutant cybrids were exposed to normoxia or 1% oxygen for 24 h as described above. In the Cytb 4.1wt cybrid, the NDUFA9 signal was mainly found in SC CI + CIII_2_ (82% NDUFA9 normalized to VDAC1), 15% was found in SC of HMW and about 3% in free CI (Figure 4B). Exposure to hypoxia did not significantly alter the NDUFA9 signal distribution but increased the levels of the UQCRC1 signal in CI + CIII_2_ and CIII_2_ in the Cytb 4.1 wt cybrids (Figure 4B,D). Unlike the RISP KO or the Pat.N fibroblasts, the Cytb 17.3E cybrid showed a strong signal with the NDUFA9 antibody (Figure 4A, NDUFA9 panel). Because the mutant Cytb 17.3E cybrid cannot assemble CIII (Figure 4A, UQCRC1 panel), there is no formation of the SC CI + CIII_2_ as observed in the Cytb 4.1wt cybrid. However, there is a faster migration of the NDUFA9 band in BN-PAGE (labeled with an asterisk) than the migration of free CI suggesting that this fast migrating band belongs to a CI assembly intermediate and not to the fully assembled CI (Figure 4A). We have recently reported the presence of this assembly intermediate in these cybrid mutant cells [38]. About 46% of the NDUFA9 signal in the Cytb 17.3E cybrid was found as CI, 41% in the subcomplex and 13% as SC of HMW. Exposure to hypoxia did not have much of an effect on the total levels of NDUFA9 signal (normalized to VDAC1) in the Cytb 17.3E cybrid. The levels of NDUFA9 in the CI band in hypoxia were slightly higher but not statistically significant (Figure 4C, *p* = 0.6) than the one observed in normoxia, suggesting a favorable condition for CI assembly at 1% oxygen.

The CA65 cybrid contains a point mutation in the mtDNA (G6930A) at a 65% heteroplasmy (65% of mutant mtDNA). This mutation generates a stop codon in the *COX1* gene that results in the production of a 170 amino acid-truncated protein leading to CIV deficiency (Figure 4E, COX1 panel) [50]. In CAwt cybrids (100% wild type mtDNA) during normoxic conditions, most of CI (NDUFA9 signal) was found in CI + CIII_2_ + CIV and CI + CIII_2_, and a very small amount (9% of the signal) was detected in SCs of HMW (Figure 4E,F). The UQCRC1 signal was distributed in SCs CI + CIII_2_ + CIV, CI + CIII_2_, CIII_2_ + CIV and free CIII (Figure 4E,H). Exposure to hypoxia did not alter the levels of NDUFA9 but caused a small decrease in the UQCRC1 signal in free CIII (Figure 4H). In contrast, in the mutant CA65 cybrids, CI was distributed in SCs of HMW, CI + CIII_2_ and free CI in a proportion of 30, 63 and 7%, respectively (Figure 4E, NDUFA9 panel and Figure 4G). Although the CA65 cybrids did not have a pleiotropic defect in CI in the absence of CIV, as we observed in our mouse CIV deficient COX10 KO fibroblasts, exposure to hypoxia increased the total levels of NDUFA9 by about 2-fold (normalized to VDAC1). Exposure of CA65 cybrids to hypoxia caused a redistribution of NDUFA9 signal into 20% in free CI, 50% in SC CI + CIII_2_ and 30% in SCs of HMW (Figure 4E,G). These results suggest that the HMW supercomplex organization is favored under low oxygen levels and, presumably, under low oxidative stress. The UQCRC1 signal in CA65 cybrids is found in the SC CI + CIII_2_ but not in the respirasome, as observed in the CAwt cells due to the lack of CIV assembly in the mutant cybrid. Hypoxia increased the total levels of UQCRC1 in SC CI + CIII_2_ by 2-fold and in free CIII by 1.5-fold (Figure 4E,I).

**Figure 5:**
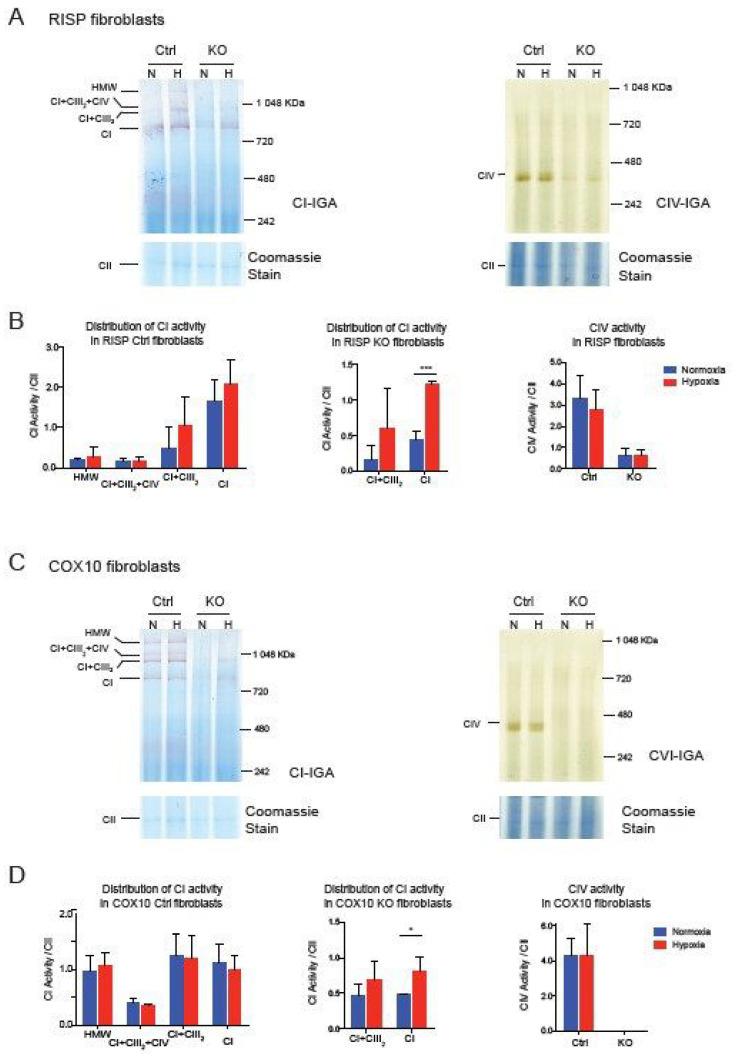
Effect of hypoxia on CI and CIV activity in RISP and COX10 fibroblasts. (**A**) Control and RISP KO lung fibroblasts were exposed to 1% oxygen for 24 h and cell processed and OXPHOS complexes separated by BN-PAGE. Then in gel activity stain (IGA) was performed with appropriate substrates for CI and CIV, respectively. After the activity stain, gels were stained with Coomassie blue to visualize all OXPHOS complexes. (**B**) Quantification of IGA for CI and CIV by densitometry using ImageJ software. Values of the activity stain were normalized to the CII band as protein loading control after gels were stained with Coomassie blue. Statistical differences were determined using Student’s *t*-test (*n* = 3). Statistical significance was represented by *** *p* < 0.001. (**C**) Control and COX10 KO skin fibroblasts were exposed to hypoxia, and IGA was performed as described in (**A**). (**D**) Quantification of IGA for CI and CIV by densitometry as described in (**B**). Statistical significance after Student’s *t*-test is represented by * *p* < 0.05 (*n* = 3).

Taken together, these results suggest that the CI pleiotropic effect observed in CIII and CIV mouse fibroblast is not shared in human cybrids, although they share similar mitochondrial respiratory deficiencies. Whether assembly of CI is pleiotropically impaired or not, exposing deficient cells to hypoxia showed a robust effect in the stability/assembly of CI. To determine if hypoxia has an effect in the enzymatic activity of CI, we performed CI and CIV in gel activity stain of BN-gels of RISP and COX10 fibroblasts (Figure 5). In RISP control lung fibroblasts, CI activity stain was observed in all different SC architectures containing CI but exposure to 1% oxygen did not significantly increase the stain (Figure 5A left panel and Figure 5B left graph). In agreement with Western blot data in Figure 1, RISP KO fibroblasts showed a significant increase in CI activity stain when exposed to hypoxia (Figure 5A left panel and Figure 5C center graph). We did not see an improvement in CIV activity stain, either in control or in RISP KO fibroblasts (Figure 5A right panel and Figure 5B right graph). In the case of the COX10 KO fibroblasts, we observed similar results with a significant increase in CI activity stain for the CI band (Figure 5C left panel and Figure 5D center graph).

### 3.3. De Novo Assembly of CI after Depletion of Mitochondrial Encoded Subunits Is Faster in Hypoxia

We investigated the de novo synthesis of mitochondrial respiratory complexes after their depletion under normoxic and hypoxic conditions. To deplete respiratory complexes, RISP KO, COX10 KO and their respective control fibroblasts were cultured for 6 days in the presence of 15 μg/mL doxycycline, a reversible mitochondrial translation inhibitor [57,58,59]. This treatment is sufficient to deplete mitochondria of their mtDNA encoded subunits. In these conditions, translation of the nuclear encoded respiratory complexes subunits is not affected [52,53]. Once doxycycline was removed from the medium, cells were placed in parallel in either normoxia or hypoxia (1% oxygen) and collected at different time points (T0, T24, T48, T72 and T96 h) after doxycycline removal. The kinetics of the assembly of respiratory complexes was analyzed by BN-PAGE followed by Western blot.

**Figure 6:**
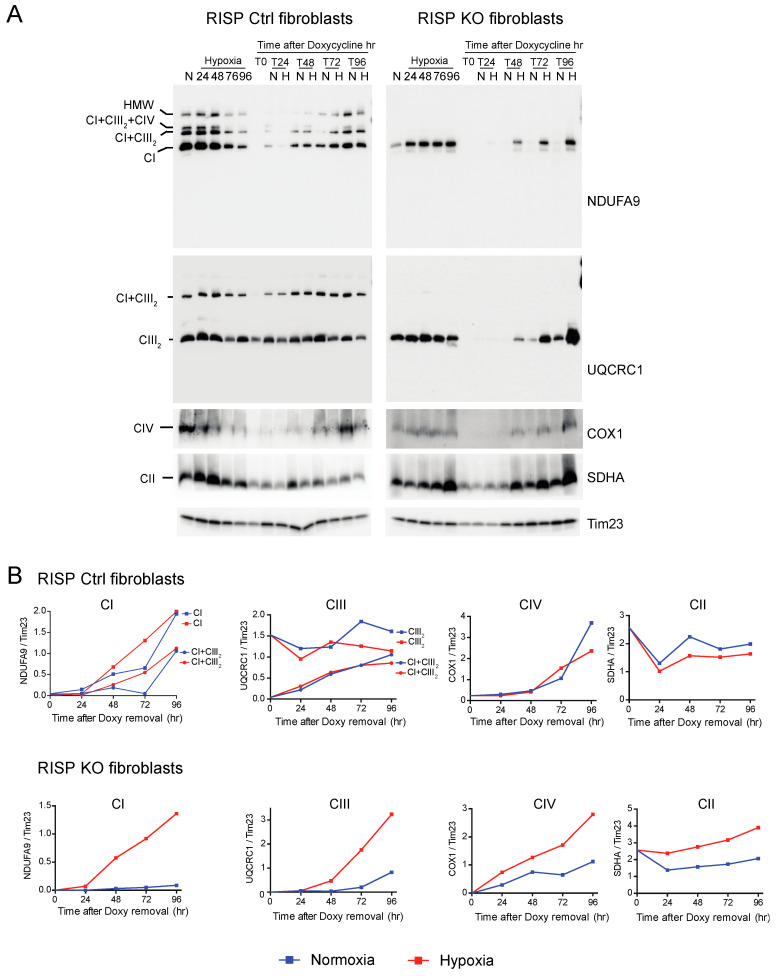
Respiratory complex and supercomplex de novo assembly in normoxia and hypoxia in RISP fibroblasts after doxycycline treatment. (**A**) RISP control and KO lung fibroblasts were treated with 15 μg/mL doxycycline for 6 days to inhibit mitochondrial protein synthesis and to deplete the organelle of respiratory complexes. The drug was removed (time 0, T0) and cells were exposed in parallel to either normoxia (N, 21% oxygen) or hypoxia (H, 1% oxygen) for different times: 24, 48, 72 and 96 h (T24, T48, T72 and T96 respectively). As a control, untreated fibroblasts were exposed to the different times to hypoxia (24 to 96 h). Respiratory complexes were extracted with digitonin and analyzed by BN-PAGE followed by Western blotting. For Ctrl fibroblasts, two identical blots were run and antibodies sequentially added in the following order, on blot 1: NDUFA9 (CI subunit) and COX1 (CIV subunit), on blot 2: UQCRC1 (CIII subunit) and SDHA (CII subunit). For KO fibroblasts, blot 1: NDUFA9, COX1 and SDHA and on blot 2: UQCRC1. Tim23 was used as loading control. (**B**) The graphs represent the kinetics of assembly of CI, CIII, CIV and CII calculated at the different time points after respiratory complexes depletion by normalizing the NDUFA9, UQCRC1, COX1 and SDHA signals to Tim23 under normoxia (blue) or hypoxia (red) from blots shown in (**A**) using ImageJ software (*n* = 1). In the RISP KO fibroblasts, de novo assembly of all respiratory complexes over time was faster in 1% oxygen when compared to normoxia. In control cells, there were no differences in assembly of respiratory complexes between normoxia and hypoxia.

**Figure 7:**
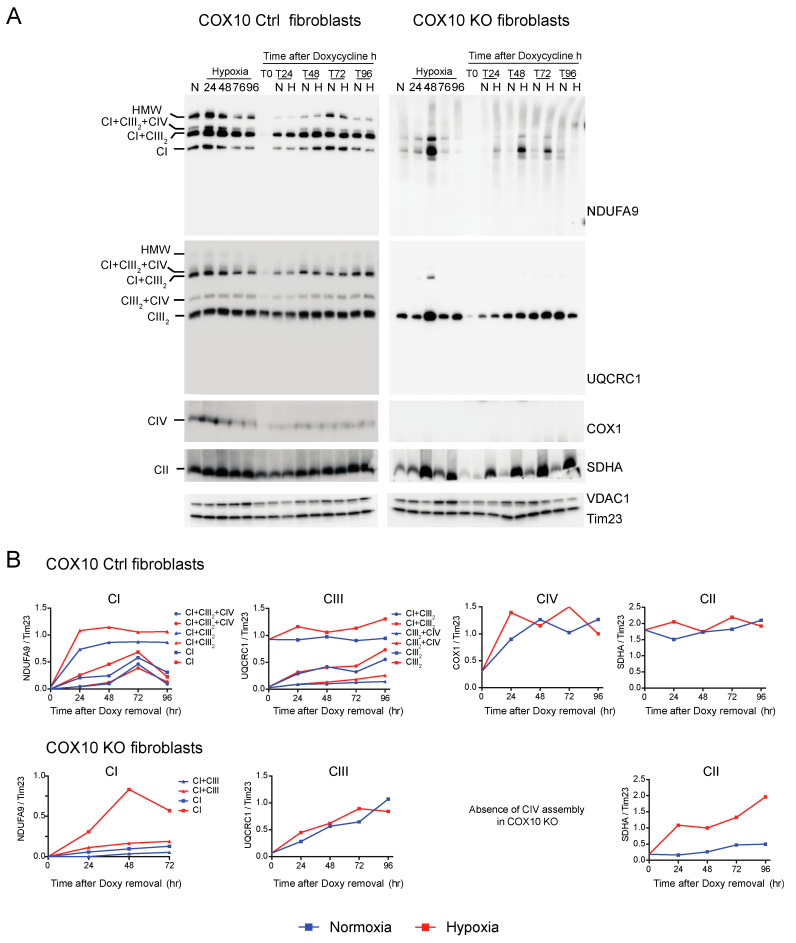
Respiratory complex and supercomplex de novo assembly in normoxia and hypoxia in COX10 fibroblasts after doxycycline treatment. (**A**) COX10 control and KO skin fibroblasts were treated with 15 μg/mL doxycycline for 6 days to inhibit mitochondrial protein synthesis and deplete the organelle of respiratory complexes. The drug was removed (time 0, T0) and cells were exposed in parallel to either normoxia (N, 21% oxygen) or hypoxia (H, 1% oxygen) for different times: 24, 48, 72 and 96 h (T24, T48, T72 and T96 respectively). As a control, untreated fibroblasts were exposed for the different times to hypoxia (24 to 96 h). Respiratory complexes were analyzed by BN-PAGE followed by Western blotting. In three identical blots, the antibodies were sequentially added in the following order, on blot 1: NDUFA9 (CI subunit), ATP5A (CV subunit) and COX1 (CIV subunit) and on blot 2: UQCRC1 (CIII subunit), and on blot 3: SDHA (CII subunit). Tim23 and VDAC1 used as loading control. (**B**) The graphs represent the kinetics of assembly of CI, CIII, CIV and CII calculated at the different time points after respiratory complexes depletion by normalizing the NDUFA9, UQCRC1, COX1 and SDHA signal to Tim23 under normoxia (blue) or hypoxia (red) from Western blots shown in (**A**) using ImageJ software (*n* = 1). In COX10 KO fibroblasts, there is no CIV assembly, so there is no graph for this complex. In the KO fibroblasts, assembly of CI and CII, but not CIII, was enhanced over time in 1% oxygen compared to the same time in normoxia. In control skin fibroblasts, no difference was observed in the de novo assembly between normoxia and hypoxia.

CI was absent in the controls as well as in RISP and COX10 KO fibroblasts after growing cells in doxycycline for 6 days (Figure 6A and Figure 7A, T0 in NDUFA9 panels). Likewise, CIV was absent in all cell lines after doxycycline treatment (Figure 6A and Figure 7A, T0 in COX1 panels). Under the same conditions, low levels of CIII still can be detected in all cell lines when compared with levels observed in normoxia (untreated) with the exception of the RISP KO fibroblasts, where CIII levels were not detected after doxycycline treatment (Figure 6A and Figure 7A, T0 in UQCRC1 panels). Although all CII subunits are encoded in the nuclear DNA, there was a decrease in the levels of CII after doxycycline treatment compared to untreated cells (Figure 6A and Figure 7A, normoxia and T0 lanes in SDHA panels). These results indicate that 6 days of doxycycline treatment were enough to almost completely deplete mitochondria from respiratory complexes CI, CIII and CIV, particularly in the RISP and COX10 KO fibroblasts. The kinetics of the newly assembled respiratory complexes and supercomplexes was followed over time (24–96 h) under normoxia or hypoxia. Figure 6 and Figure 7 show that, in mutant cells, all respiratory complexes assembled faster at 1% oxygen than at 21% oxygen. Quantification of the kinetics of assembly over time in Figure 6B shows no differences between normoxia and hypoxia of assembly of the respiratory complexes and SCs in the RISP control lung fibroblasts. In the RISP KO fibroblasts, the kinetics of the de novo assembly of all respiratory complexes, even CII, was faster in hypoxia (Figure 6B). Similar results were observed for the COX10 fibroblasts (Figure 7B). In the control COX10 skin fibroblasts, the assembly of respiratory complexes and SCs was independent of oxygen levels. In the COX10 KO fibroblasts, the assembly of CI, SC CI + CIII_2_ and CII was faster in hypoxia; however, the assembly of CIII was not affected by the oxygen concentration (Figure 7B).

### 3.4. Hypoxia Did Not Increase ROS in RISP and COX10 Fibroblasts

The generation of ROS upon hypoxia exposure has been a controversial topic. We hypothesized that part of the mechanism of destabilization of CI in the CIII and CIV deficient fibroblasts was related to increased levels of free radical production by the impaired ETC and when cells were exposed to hypoxia the production of free radicals was normalized contributing to the stability of CI [34,35]. Previously, using H_2_DCFDA (2′,7′ dichlorodihydrofluorescein diacetate) fluorescent dye, we were unable to observe any ROS production in the RISP KO fibroblast exposed to 1% oxygen for 24 h [35]. Here, we used the radiometric biosensor RoGFP to follow changes in redox state in the RISP and COX10 KO fibroblasts exposed to hypoxia. We used the RoGFP probe targeted either to the mitochondrial matrix (Matrix-RoGFP) or to the intermembrane space (GDP-RoGFP) [54]. The ratio of the fluorescence emitted (captured at 510 nm) by excitation at two wavelengths (405 and 495 nm) is proportional to the ROS generated. High 405/495 ratio indicates oxidation of the biosensor [54].

**Figure 8:**
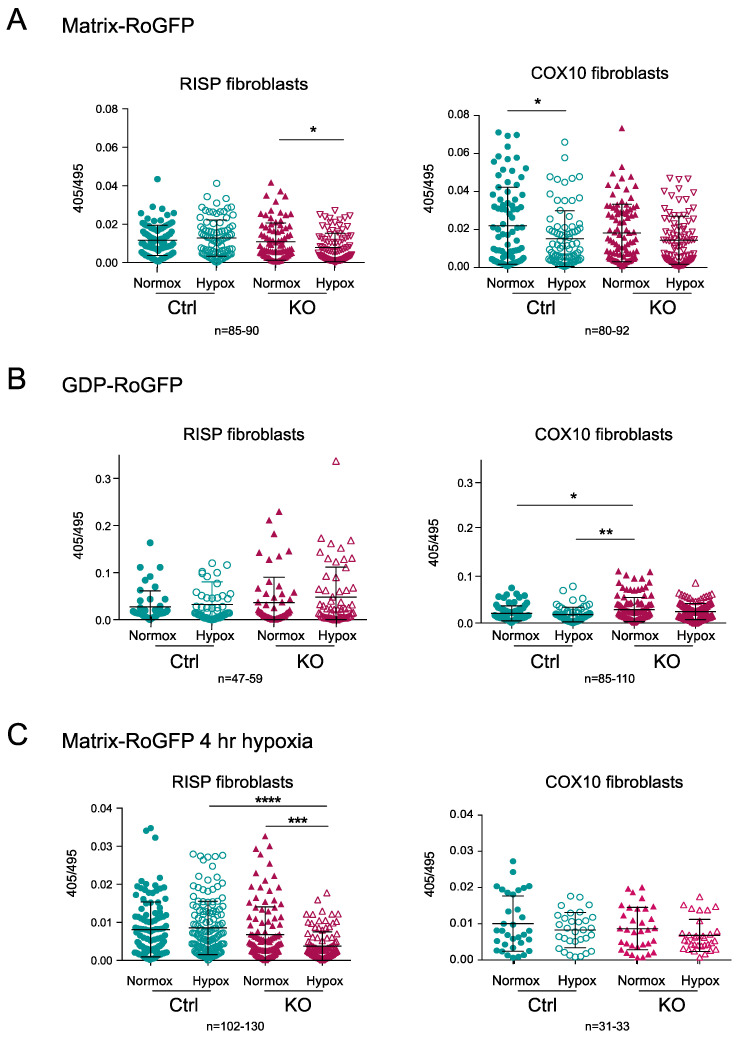
Determination of mitochondrial ROS production using the redox sensitive biosensor RoGFP in RISP and COX10 KO fibroblasts. (**A**) Controls, RISP and COX10 KO fibroblasts were transfected with plasmids expressing Matrix-RoGFP (targeted to the mitochondrial matrix) and 24 h after transfection cells were placed either under normoxia (21% oxygen) or hypoxia (1% oxygen) for 24 h. Cells were fixed with 4% PFA and imaged in a confocal microscope at two excitation wavelengths, 405 and 495 and emission of 510 nm. Fluorescence ratio 405/495 was calculated using ImageJ software for each cell. (**B**) Controls, RISP and COX10 KO cells were transfected with GDP-RoGFP (targeted to the mitochondrial intermembrane space) and exposed to normoxia and hypoxia as described in (**A**). (**C**) Controls, RISP and COX10 KO fibroblasts were transfected with matrix-RoGFP and 24 h post-transfection exposed to either normoxia or 1% oxygen for 4 h and immediately fixed with 4% PFA and imaged as described in (**A**). Graphs represent the combined results of the fluorescence of individual cells for at least 3–4 independent experiments. In each experiment, 20 to 30 cells were analyzed. Each symbol represents one cell and the number of cells (*n*) is indicated below each graph. Statistical analysis was performed using one-way ANOVA followed by Tukey’s multiple comparisons test. Statistical significance is represented by * *p* < 0.05, ** *p* < 0.01, *** *p* < 0.001 and **** *p* < 0.0001. Exposure to hypoxia did not increase ROS production in any of the cell lines.

The matrix-RoGFP biosensor detected a significant decrease in the 405/495 ratio in the RISP KO fibroblasts exposed to hypoxia compared to normoxia, indicating lower ROS production at 24 h in 1% oxygen (Figure 8A); however, in the control RISP cells, there was no change in ROS production between normoxia and hypoxia. In COX10 control fibroblasts, there was a significant decrease in ROS production when cells were exposed to hypoxia, but no difference was observed in the COX10 KO fibroblasts (Figure 8A). The intermembrane space GDP-RoGFP biosensor did not show any significant differences in the ROS production between normoxia and hypoxia exposure in both control and RISP KO fibroblasts (Figure 8B). The COX10 KO fibroblasts showed significantly higher 405/495 ratio in the intermembrane space when compared to control cells in normoxia, but hypoxia did not increase ROS levels when compared to normoxia in these fibroblasts (Figure 8B). Because many studies only measure ROS production in short time periods of low oxygen exposure, we measured changes in the 405/495 nm fluorescence after 4 h of 1% oxygen exposure. We did not observe any increase in ROS production but rather a significant decrease in the oxidation of matrix-RoGFP in the RISP KO cells when exposed to 1% oxygen for 4 h (Figure 8C). In summary, hypoxia did not increase RoGFP oxidation in controls, RISP or COX10 KO fibroblasts, supporting our hypothesis of low ROS production at low oxygen.

### 3.5. Dependence of HIF-1α on the Stability of CI and Respiratory Complexes

Since hypoxia favored the assembly and stability of respiratory complexes, particularly of CI in the RISP and COX10 KO fibroblasts, we investigated whether this was dependent on the expression of HIF-1α. This hypoxic transcription factor subunit is constantly targeted for degradation by prolyl hydroxylases (PHD) under normoxic conditions. Low oxygen or pharmacological inhibition of PHD results in an accumulation of HIF-1α. Some studies indicated that deletion of RISP and cytochrome c inhibited HIF-1α accumulation in hypoxia (reviewed in [60]).

**Figure 9:**
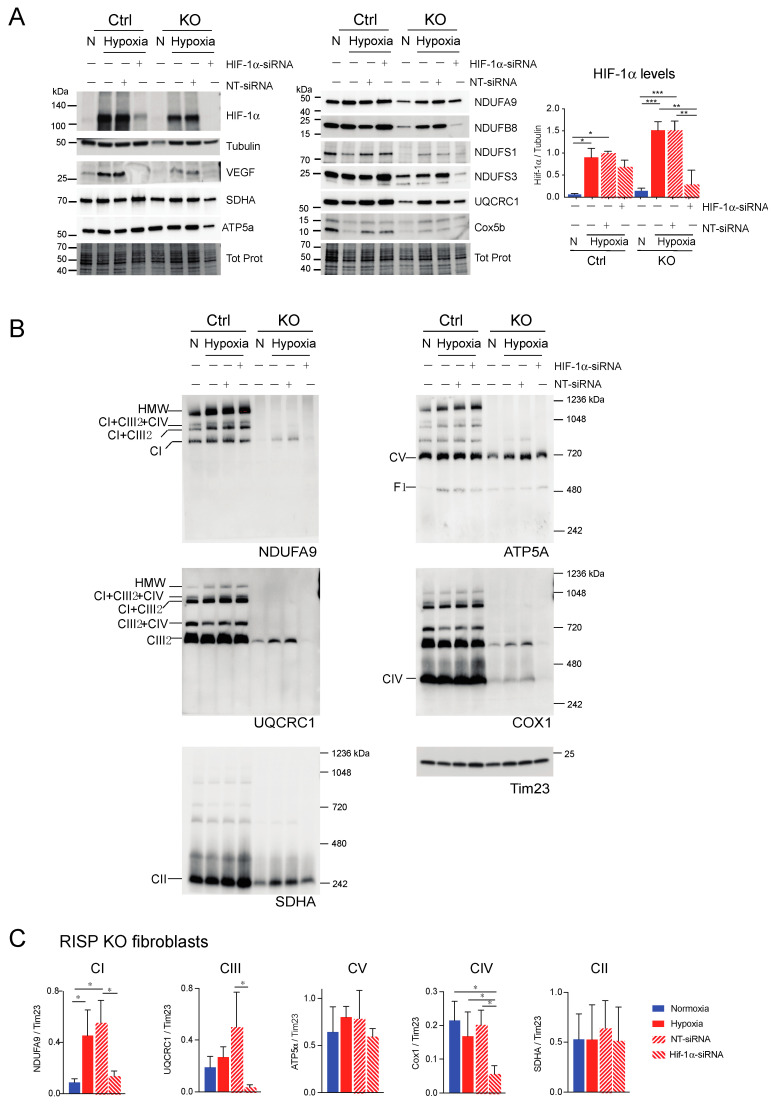
Effect of HIF-1α siRNA on stability of respiratory complexes in RISP KO fibroblasts. RISP control and KO lung fibroblasts were transfected with either a mixture of oligonucleotides against HIF-1α (HIF-1α siRNA) or non-targeted oligos (NT-siRNA) as control. Two rounds of transfection were performed to achieve a robust silencing. (**A**) After 120 h transfection with oligos, cells were exposed to normoxia (N) or hypoxia (H, 1% oxygen) for 24 h and steady-state levels of HIF-1α, VEGF, and subunits of respiratory complexes SDHA, ATP5a, NDUFA9, NDUFB8, NDUFS1, NDUFS3, UQCRC1 and Cox5b were determined by SDS-PAGE. Loading controls are represented by tubulin and total protein in the blots (representative blots) using the stain-free technology. The graph shows the densitometric analysis of the levels of HIF-1α normalized to tubulin using ImageJ software (*n* = 2). Statistical differences were determined using one-way ANOVA followed by Tukey’s multiple comparisons test. Statistical significance is represented by * *p* < 0.05, ** *p* < 0.01 and *** *p* < 0.001. Blue bars represent normoxia, red bars represent 1% oxygen, upward hatched lines represent siRNA non target control and cells exposed to 1% oxygen and downward hatched lines represent siRNA of HIF-1α. (**B**) Effect of HIF-1α siRNA on the assembly of OXPHOS complexes was assessed by BN-PAGE. In two identical blots, the antibodies were sequentially added in the following order, on blot 1: NDUFA9, ATP5A and COX1 and on blot 2: UQCRC1 and SDHA (CII subunit). Tim23 was used as a loading control. (**C**) Quantification of the levels of OXPHOS complexes in the RISP KO fibroblasts. Western blot signals of BN-PAGE for each complex were analyzed using ImageJ and values normalized to Tim23 mitochondrial loading control (*n* = 2–4). Statistical differences were determined using one-way ANOVA followed by Tukey’s multiple comparisons test. Statistical significance is represented by * *p* < 0.05. Hypoxia increased the steady-state levels of HIF-1α, VEGF, and subunits of respiratory complexes compared to normoxic levels and the effect was abrogated by silencing the transcription factor. The stability of CI, CIII and CIV in hypoxia was dependent on HIF-1α expression.

**Figure 10:**
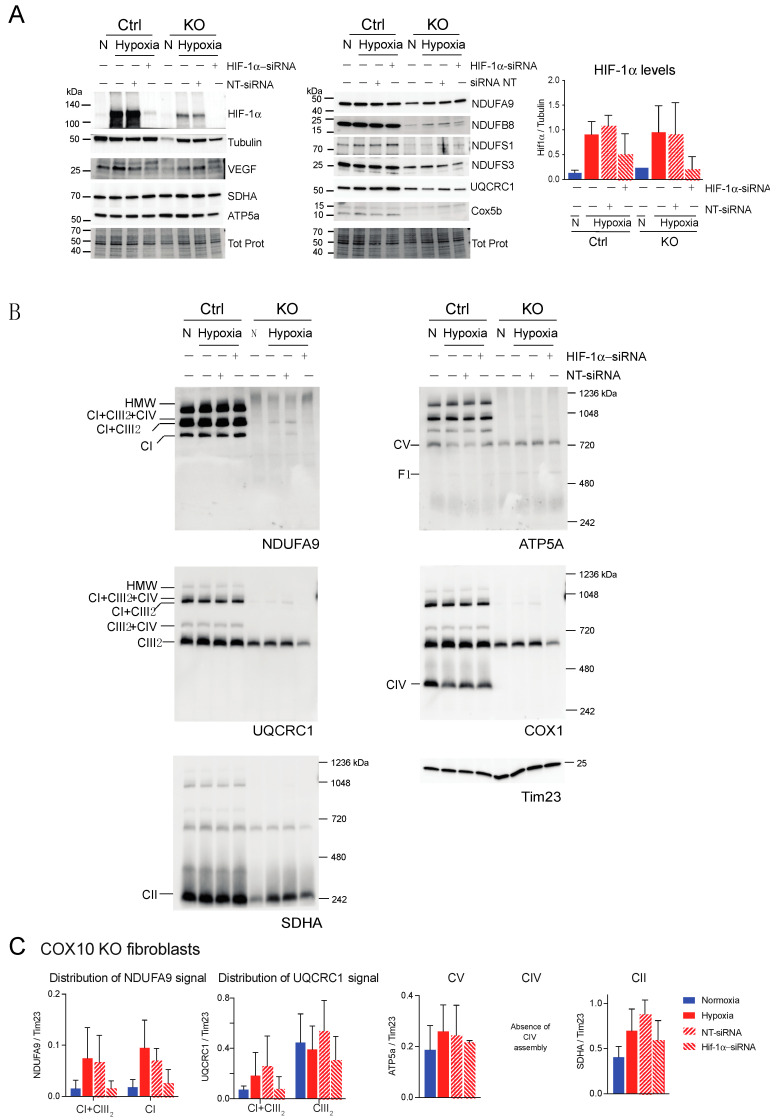
Effect of HIF-1α siRNA on stability of respiratory complexes in COX10 fibroblasts. COX10 control and KO skin fibroblasts were transfected with either a mixture of oligonucleotides against HIF-1α (HIF-1α siRNA) or non-targeted oligos (NT-siRNA) as control. Two rounds of transfection were performed to achieve a robust silencing. (**A**) After 120 h transfection with oligos, cells were exposed to normoxia (N) or hypoxia (H; 1% oxygen) for 24 h and steady-state levels of HIF-1α, VEGF, and subunits of respiratory complexes SDHA, ATP5a, NDUFA9, NDUFB8, NDUFS1, NDUFS3, UQCRC1 and Cox5b were determined by SDS-PAGE. Loading controls are represented by tubulin and total protein in the blots (representative blots shown) using the stain-free technology. Graph shows the densitometric analysis of the levels of HIF-1α normalized to tubulin using ImageJ software (*n* = 2). Blue bars represent normoxia, red bars represent 1% oxygen, upward hatched lines represent siRNA non-target control and cells exposed to 1% oxygen and downward hatched lines represent siRNA of HIF-1α of cells exposed to 1% oxygen. (**B**) The effect of HIF-1α siRNA on respiratory complexes was assessed by BN-PAGE. In two identical blots, the antibodies were sequentially added in the following order on blot 1: NDUFA9 (CI subunit), ATP5A (CV subunit) and COX1 (CIV subunit) and on blot 2: UQCRC1 (CIII subunit) and SDHA (CII subunit). Tim23 was used as a loading control. (**C**) Quantification of the levels of OXPHOS complexes in COX10 KO skin fibroblasts. Western blots of BN-PAGE for each complex were analyzed using Image J and values normalized to Tim23 mitochondrial loading control (*n* = 2–4). Statistical differences were determined using one-way ANOVA. Blue bars represent normoxia, red bars represent 1% oxygen, upward hatched lines represent siRNA non-target control of cells exposed to 1% oxygen and downward hatched lines represent siRNA of HIF-1α of cells exposed to 1% oxygen. Stability of CI in hypoxia was dependent on HIF-1α expression and the levels of CIII decreased with HIF-1α silencing.

We investigated whether the RISP and COX10 KO cells accumulated HIF-1α under low oxygen tension (Figure 9A and Figure 10A, respectively). Both RISP and COX10 KO fibroblasts were able to stabilize HIF-1α when exposed to 1% oxygen for 24 h (Figure 9A and Figure 10A, HIF-1α panel and graph). In control and RISP KO lung fibroblasts, there was a 13- and a 10-fold significant increase in the levels of HIF-1α, respectively (normalized to tubulin), when cells where exposed to hypoxia compared to normoxia (Figure 9A graph). In the COX10 control skin fibroblasts, there was a 7-fold increase in the levels of HIF-1α (normalized to tubulin) in cells exposed to 1% oxygen compared to normoxia, whereas in the COX10 KO fibroblasts, there was only a 4-fold increase in the transcription factor when cells were exposed to hypoxia (Figure 10A HIF-1α panel and graph). We observed that as hypoxia stabilized HIF-1α in all cell lines, it also increased the steady-state levels of VEGF, one of the targets of this transcription factor (Figure 9A and Figure 10A).

Next, we explored if HIF-1α was required for the stability of CI and other respiratory complexes during hypoxia in RISP and COX10 KO fibroblasts. Using a mixture of Stealth RNAi duplex oligoribonucleotides against mouse HIF-1α, we transfected cells to knockdown HIF-1α. After 6 days and 2 rounds of transfection, we observed a complete knockdown of HIF-1α when cells were exposed to low oxygen, particularly in the RISP and COX10 KO fibroblasts (Figure 9A and Figure 10A, respectively). In control cells, the silencing in hypoxia was less efficient and low levels of HIF-1α remained (about 8–20% of non-silenced levels). As expected, the non-targeted oligoribonucleotides (NT-siRNA) did not interfere with the stabilization of HIF-1α by hypoxia in any of the cell lines and levels of VEGF were increased when HIF-1α accumulated (Figure 9A and Figure 10A). We also examined the steady state level of various subunits of the respiratory complexes and observe that the levels of CI subunits (NDUFA9, NDUFB8, NDUFS1 and NDUFS3) were low in the KO fibroblasts compared to controls. When KO fibroblasts were exposed to hypoxia, even in the presence of the NT-siRNA, there was an increase in the steady-state levels of CI subunits. HIF-1α silencing abrogated the increase in the levels observed in hypoxia, particularly in the RISP KO fibroblasts (Figure 9A). Similar results were observed for subunits of CII (SDHA), CIII (UQCRC1) and CIV (Cox5b) (Figure 9A).

BN-PAGE and Western blot analysis revealed that in the absence of HIF-1α, CI was dramatically decreased in both RISP and COX10 KO fibroblasts, even when cells were exposed to 1% oxygen (Figure 9B,C and Figure 10B,C; statistical significance only for RISP KO cells in Figure 9C). KO fibroblasts transfected with NT-siRNA oligos were able to stabilize CI when exposed to hypoxia (Figure 9B and Figure 10B, NDUFA9 panels). Knockdown of HIF-1α in control fibroblasts did not have any effect in respiratory complexes. In the RISP KO fibroblast, silencing HIF-1α also significantly affected the assembly of CIII and CIV but not CII and CV when compared to the NT-siRNA control (Figure 9B,C). In the COX0 KO fibroblasts, silencing of HIF-1α resulted in a small reduction in CII and CIII levels but CV was not affected when compared to the NT-siRNA under hypoxia (Figure 10B,C). Taken together, these results suggest that the beneficial effect of hypoxia in the stability/assembly of respiratory complexes in the defective RISP and COX10 KO cells is mediated by HIF-1α.

## 4. Discussion

An interdependence of respiratory complexes has been observed in many cases. We and others have shown that in CIII- and CIV-deficient cells, there is a secondary defect in CI [39,40,42,43,61]. Interestingly, we discovered that when RISP and COX10 KO fibroblasts were exposed to hypoxia, this pleiotropic effect on CI was reduced. We proposed that an increase in ROS was one of the culprits causing CI instability in the CIII- and CIV-deficient cells [34,35]. In the present study, we investigated the mechanism downstream of mitochondrial ROS production that could explain the stability/assembly of CI. Our results clearly show that the stability/assembly of CI in RISP and COX10 deficient fibroblasts was dependent on the accumulation of HIF-1α at low oxygen levels. In fact, in the RISP KO fibroblasts, both CIII and CIV stabilization/assembly also required the presence of HIF-1α during hypoxia.

HIF-1α is one of the subunits of the hypoxia-inducible transcription factor 1 (HIF-1), which is constantly being synthesized and degraded under high oxygen tension (21% oxygen or normoxia) by the combined action of the prolyl hydroxylases (PHDs), which hydroxylates HIF-1α, and the von Hippel–Lindau protein (pVHL), a ubiquitin E3 ligase that recognizes the hydroxylated HIF-1α and targets it for proteasomal degradation [62]. PHDs require oxygen for activity, so in hypoxia HIF-1α hydroxylation does not occur, and the protein is not degraded. Once HIF-1α accumulates, it is able to dimerize with the HIF-1β subunit forming an active HIF-1 that binds to hypoxia response elements in the nuclear genome to initiate low oxygen-dependent transcriptional programs [63]. HIF-1 activation leads to a downregulation of mitochondrial respiration and to an increase in glycolysis. HIF-1 increases the expression of pyruvate dehydrogenase kinase [64], which inhibits the activity of the pyruvate dehydrogenase complex (PDC). Inhibition of PDC causes an increase in anaerobic glycolysis by preventing the catabolism of pyruvate to acetyl CoA, which feeds into the tricarboxylic acid cycle and the electron transport chain [64]. In addition, HIF-1 regulates CIV activity by switching COX4-1 to the COX4-2 subunit isoform, associated with a more efficient enzyme in low oxygen conditions [65].

Studies indicated that the activation of the hypoxia transcriptional program requires mitochondrial ROS [60]. At low levels, ROS act as signaling molecules, but when in excess ROS produce detrimental damage and oxidative stress. Mitochondria is the cellular compartment where most of the ROS are produced and CI and CIII are the major contributors of free radicals [32]. The production of ROS in hypoxia has been a matter of controversy for many years among investigators (reviewed in [66,67]). The discrepancies have been attributed to methodology, cell types, time in hypoxia and other variants. Work by Waypa and colleagues tried to clarify this controversy using a redox sensitive GFP (RoGFP) targeted to different subcellular compartments [54]. They found that in smooth muscle cells perfused with hypoxic media (1.5% oxygen for 30 min), there was an increase in the oxidation of RoGFP when targeted to the cytosol or the mitochondrial intermembrane space indicating increased ROS production. However, when RoGFP was targeted to the mitochondrial matrix, its oxidation decreased in hypoxia [54,68]. Using the same ROS biosensors in our RISP and COX1O KO fibroblasts, we observed a significant decrease in the oxidation of matrix-RoGFP in RISP KO lung fibroblasts and in COX10 control skin fibroblasts in hypoxia in agreement with Waypa’s reports. However, unlike their studies, we did not observe an increase in the oxidation of GDP-RoGFP in hypoxia in any of the cells investigated. This difference could be due to differences in cell type or in time points used as they measured changes in RoGFP fluorescence for 30 min in hypoxia (1.5% oxygen), whereas our studies were done after 4 or 24 h at 1% oxygen.

Early work by Schumacher’s group revealed that ROS produced by CIII was required to stabilize HIF-1α in hypoxia in Hep3B and 293 cells [69]. Moreover, they showed that RISP was responsible for the ROS signaling and was required for HIF-1α stabilization [70,71,72,73], and proposed RISP as an oxygen sensor regulating cellular responses during hypoxia [74]. Refining their knockdown studies, they produced conditional knockout of floxed RISP in pulmonary artery smooth muscle cells and showed that deletion of the CIII subunit abrogated the oxidation of cytosolic and GDP-RoGFP, whereas the matrix-RoGFP remained unchanged during hypoxia [68]. Contrasting evidence has been obtained by other groups. Vaux et al. found that HIF-1α was stabilized without a functional electron transport chain using various cell lines [75]. Chua et al. showed that HIF-1α stabilization was independent of ROS production and proposed that the cellular response to hypoxia can be regulated by altering mitochondrial respiration, which in turn will regulate the cellular oxygen availability required for HIF-1α [76]. Our results agree with Vaux [75], Chua [76] and others. We observed that the accumulation of HIF-1α after exposure to 1% oxygen was conserved after deletion of RISP and COX10 genes. Additionally, as mentioned above, we did not observe an increase in ROS production when exposing these fibroblasts to hypoxia. Interestingly, HIF-1α levels are also regulated by other mechanisms where ROS does not seem to be the major driver [77]. Shvetsova et al. found that cells deficient in SOD2 or mitochondrial enoyl CoA reductase displayed reduced levels of HIF-1α when exposed to hypoxia, whereas cells deficient in Mpv17 showed increased levels of the transcription factor, even at normoxia. These mitochondrial defects appear to exert a distinct regulation of HIF-1α levels at the protein degradation and transcriptional level rather than a ROS-mediated regulation [77].

Our studies showed that low oxygen levels reduced the pleiotropic CI defect in various cells defective in CIII and CIV, but whether the CI pleiotropic defects are due to an impaired assembly or to a decreased stability requires further analysis. CI is the largest complex of the ETC, with 45 subunits. CI assembly is very intricate and the complete process is still elusive. Numerous studies indicate that CI assembly occurs in a modular, rather than a sequential fashion. CI assembly modules include the N, Q, Q/ND1, ND2 and ND4 modules and it has been proposed that in the assembly process, the N module is the last one to be incorporated [78]. Our results using the Pat.N fibroblasts and in the Cytb 17.3E cybrids showed the presence of CI assembly intermediates. In the case of Pat.N, the assembly intermediate of >720 kDa (asterisk Figure 3A NDUFA9 panel) is observed at 1% oxygen and at higher oxygen levels the NDUFA9 signal is found in the respirasome. Because this assembly intermediate is only observed when cells are exposed to 1% oxygen and not in normoxia, it suggests that the CYC1 mutation in Pat.N causes an impaired assembly that is bypassed by low oxygen. In the case of the Cytb 17.3E cybrids, a fast migrating CI band is observed in normoxia, suggesting the stalling of the last intermediary into the assembly process. This assembly intermediate is most likely the 830 kDa intermediate we have described by functional analysis and comprehensive proteomics [38]. The absence of CIII in Cyb17.E cybrids blocks the assembly of CI, preventing the incorporation of the N module [38].

In the case of the RISP and COX10 KO fibroblasts, we did not observe any assembly intermediates either in normoxic or hypoxic conditions. The faster kinetics of de novo assembly of respiratory complexes in hypoxia after doxycycline removal suggest a defective assembly mitigated by low oxygen. It is possible that the faster kinetics of assembly observed during hypoxia are due to very low levels of mitochondrial ROS when there is a complete absence of or partially assembled ETC. However, further studies are required to determine if the expression of assembly factors or chaperones is enhanced at low oxygen levels, promoting assembly. In fact, the SC yeast assembly factor Rcf1 and its mammalian orthologs HIGD1 and HIGD2 belong to hypoxia-induced gene 1 protein family [14,15,16]. HIG2D gene expression is regulated by hypoxia and glucose availability [22].

In this study, we showed the importance of HIF-1α to abrogate the pleiotropic defect in CI. The activation of the HIF-1 transcriptional program induces the expression of various genes related to energy metabolism. To adapt to the low oxygen availability, there is an activation of glycolysis and inhibition of the aerobic mitochondrial metabolism [47,63]. Elegant studies by Mootha’s group revealed in a genome-wide CRISPR/Cas9 screening of cells treated with antimycin (CIII inhibitor) that pVHL was the top candidate in suppressing the mitochondrial dysfunction [45]. The initial discovery led them to the striking finding that when a CI-deficient mouse, the *Ndufs4*^−/−^, was exposed to chronic hypoxia of 11% oxygen, their life span was significantly extended from ~60 to ~270 days. Moreover, the disease phenotype was ameliorated even if the genetic defect still existed [45,46]. More recently, this group tested if the genetic activation of the hypoxic response could ameliorate the phenotype of the *Ndufs4*^−/−^ mouse. They found that when crossing the CI deficient mouse with various mice lacking PHDs or mouse with a point mutation in the pVHL it was not sufficient to prevent disease phenotype [79]. However, their experiments cannot exclude the importance or the involvement of HIF-1α in the in vivo rescue of the disease phenotype by chronic hypoxia. Unfortunately, in all these studies, CI assembly was not investigated in tissues of the *Ndufs4*^−/−^ mouse exposed to chronic hypoxia. Initial characterization of CI assembly in *Ndufs4*^−/−^ mouse tissues showed the presence of 200 and 830 kDa intermediates, 830 + CIII_2_ and CI + CIII_2_ bands [44]. The 200 kDa and the CI + CIII_2_ retained dehydrogenase activity [44]. The same assembly intermediates were confirmed in immortalized *Ndufs4*^−/−^ mouse embryonic fibroblasts [80].

Our results are in contrast with the recent report in human leukemia monocytes that showed a decrease in CI levels when exposed cells were exposed to hypoxia. The CI decrease was caused by the degradation of TME126B, a CI assembly factor [81]. The degradation of TEM126B was dependent on HIF-1α and silencing the hypoxic transcription factor attenuated the degradation of CI. The differences observed with our findings could be related to different regulatory mechanisms in cancerogenic cell types vs fibroblasts.

The exact mechanism of how mitochondrial dysfunction is benefited by the activation of the hypoxia transcriptional program through HIF-1α, particularly for the assembly/stability of CI, is still unknown and further studies should focus on identifying factors which are involved in this process. Our findings provide some mechanistic insights on how hypoxia is beneficial to the *Ndufs4^−/−^* mouse. However, recent studies by Jain and collaborators using CRISPR screening indicated that the transcriptional program induced by hypoxia is intricated and involves many genes not previously connected to HIF [82]. Interestingly, in their KO screen, they found that CI-related growth defects, but not other respiratory complex defects, were “buffered” by 1% oxygen [82].

Understanding the role of hypoxia, HIF-1α and downstream targets on stability/assembly of the ETC could be advantageous to develop new therapeutic targets amenable to pharmacological inhibition. Some of the emerging potential therapies to treat mitochondrial disorders include hypoxia [83]. However, implementation of chronic hypoxia in the clinics could be difficult and long-term physiological consequences could develop [84].
